# Organic food as a healthy lifestyle: A phenomenological psychological analysis

**DOI:** 10.3402/qhw.v8i0.20559

**Published:** 2013-06-14

**Authors:** Elisabeth Von Essen, Magnus Englander

**Affiliations:** 1 Department of Work Science, Business Economics and Environmental Psychology, Swedish University of Agricultural Sciences, Alnarp, Sweden; 2 Department of Social Work, Malmö University, Malmö, Sweden

**Keywords:** Health psychology, qualitative method, well-being, nature

## Abstract

This study explored the phenomenon of the lived experience of choosing a healthy lifestyle based upon an organic diet as seen from the perspective of the young adult. Interviews were collected in Sweden and analyzed using the descriptive phenomenological psychological research method. The results showed the general psychological structure of the phenomenon, comprising four constituents: (1) the lived body as the starting point for life exploration, (2) a narrative self through emotional-relational food memories, (3) a conscious life strategy for well-being and vitality, and (4) a personal set of values in relation to ethical standards. The results provide plausible insights into the intricate relation between psychological meaning and the natural world.

Choosing a lifestyle based upon an organic diet seems to be a growing trend among young adults^1^ in Western society. As a consequence, the choice of a particular diet can be seen as telling a story about a person and indicating how one wants to be identified by others (Bisogni, Connors, Devine, & Sobal, 2002). In general, a person's identity, in relation to his or her choice of diet, can be seen as being dependent on early experiences of food, in terms of trajectories, such as persistent thoughts, feelings, strategies, and actions (Bisogni, Jastran, Shen, & Devine, 2005; Devine, 2005; Devine, Connors, Bisogni, & Sobal, 1998). This makes the young adult's choice of a particular diet a way to connect and to belong to a certain social group as well (Fischler, 1988; Hunt, Fazio, MacKenzie, & Moloney, 2011). Therefore, choosing a lifestyle based upon an organic diet could suggest an opportunity to establish a sense of stability for the young adult, in terms of identity and belonging. Nevertheless, trajectories and choice of diet can change during a person's lifespan, especially in relation to dramatic life events created by the interplay between relationships, contexts, and environments (Devine et al., 1998). In addition, Bisogni et al. (2002) point out that people can have different identities related to their diet and see the relation between them as a continuous, reflective evaluation of themselves. Hence, the young adult's choice of a healthy diet means a lot more than just feeding the right type of nutrition into one's body. Such a commitment also communicates to others an experiential relation between the self and the natural world. The aim of this particular study was to further explore young adults’ experiences of a healthy lifestyle choice based upon an organic diet in order to discover general psychological meanings.

What then constitutes a healthy diet? Researchers taking an expert, medical approach usually claim that the key to promoting health-related behavior is to focus on the concept of nutrition (Bisogni et al., 2002). Others see healthy eating as maintaining a diet in which unnecessary additives are excluded (Halkier, 2001; Harrison & Jackson, 2009). Healthy food has also been interpreted in relational and context-related ways, for example as something that is cooked at home together with one's family (Neumark-Sztainer, Story, Ackard, Moe, & Perry, 2000; Sylow & Holm, 2009). Other researchers see healthy diets as a cultural responsibility, in order to promote health and quality of life for society at large (Bisogni, Jastran, Seligson, & Thompson, 2012). The social aspects of diets are nothing new. They have always been an essential part of certain religious, spiritual, and other cultural practices. Certain foods (e.g., vegetables) have also been interpreted as a way to take care of the body and to achieve vitality (Allicock, Sandelowski, DeVellis, & Campbell, 2008). By turning the focus from the rhetoric of “what to eat and what not to eat” in relation to the empirical aspects of the body, to concepts such as vitality and social responsibility, suggests a marked epistemic shift of the scientific study of a healthy diet. Investigating the conception of a healthy diet from the perspective of the young adult, some researchers have emphasized that they “defined health as an act of doing, and more specifically as doing the ‘right’ thing in terms of some type of physical act (e.g., healthy eating or exercise)” (Woodgate & Leach, 2010, p. 1176). To accentuate, there is more to a healthy diet for the young adult than just objectively picking out the right amount of nutrients. A healthy diet also seems to constitute a search for values and a lifestyle that portrays to others their identity and belonging.

Organic food as a healthy way of eating has been a target for consumer studies in recent years as a result of its increasing popularity (Hjelmar, 2011; Magnusson, Arvola, Hursti, Åberg, & Sjöden, 2003). For example, one study showed that those who prefer organic food as constituting their lifestyle “have a life philosophy where food plays a central role for subjectivity” (Pellegrini & Farinello, 2009, p. 959). In this particular study, the participants felt that they had more responsibility for their own well-being, tended to be more reflexive and autonomous, and used more unconventional healing practices when forced to make decisions concerning their health (Pellegrini & Farinello, 2009). Other studies addressed psychological components, such as the appreciation of the taste of organic food, the experience of well-being, and being able to contribute to a better future for themselves (Köpke, 2005). Michaelidou and Hassan (2008) even found evidence for the organic consumer having a strong sense of ethical self-identity, whereas Schifferstein and Ophuis (1998) summed up the choice of an organic diet as a way of life. In a study by Stobbelaar et al. (2007), adolescents reported that they viewed organic food as both healthy and environmentally friendly, hence indicating meanings in relation to this particular diet that went far beyond its nutritional index. Even though consumer studies could provide us with some valuable insights into the young adult's lifestyle based upon an organic diet, a human scientific study seeking the psychological meaning from the depths of the lived experience is also needed in order to provide an alternative to the consumer perspective.

The young adult's choice of a healthy lifestyle based upon an organic diet might indicate a motivation to return to the natural world. The relation between the self and the natural world is a complex one, and it has, in general, been neglected by mainstream psychological research. Adams (2005) writes,

Psychologists often emphasize that our relations with others may bring forth health or pathology, for both our self and others. Likewise, ecopsychology research is revealing that this is also true in our relations with the natural world. Human well-being and the well-being of the natural world are mutually dependent. Thus it may seem strange that until recently the discipline of psychology has mostly ignored our relationship with the rest of nature. (p. 269)

The field of ecopsychology is thus trying to provide insight into this particular gap in psychological research by providing insight into the relation between self and the natural world. Adams (2005), supportive of an ecopsychology, suggests a phenomenological position that draws heavily on the work of, for example, existential-phenomenological philosopher Maurice Merleau-Ponty. The ontological position of Merleau-Ponty (1962, 1964) points to humans as a part of the natural world through their lived body as opposed to the abstract, material body, which is the object for the natural sciences. From such an existential-phenomenological philosophical position, one could describe the essence of choosing a lifestyle based upon organic food as a fundamental way of relating to one's own nature. The choice of an organic diet could perhaps mean a return to nature and thus to our being. Furthermore, it could indicate a choice to not participate in the attempts to take control over the natural world and to treat it as if it was secondary to our own nature. As seen from such an ontological position, organic food is more like us, that is, it is part of our nature.

Leaving this theoretical position aside, there is also a need to understand the psychological meaning of young adults’ lifestyle choice to maintain an organic diet. A phenomenological human scientific study could reveal, for example, the psychological aspects (e.g., the motivations, emotions, thoughts and fantasies, struggles, and/or relational complexities) of the meaning of the phenomenon. To explore what it is like for the young adult to experience this particular phenomenon could help us to see the psychological aspects of well-being and health from the position of the lived experience and our relation to the natural world. Thus, there is also a need to stress the importance of the phenomenological human scientific level that could grant us access to psychological meanings that thrive in the life-world as described by those persons who directly experience the phenomenon. Consequently, and as stated here, the purpose of the study was to explicate and to describe the psychological meaning of young adults’ experience of choosing a healthy lifestyle based upon an organic diet.

## Method

### Participants and data collection procedures

The data for this particular study were selected from a previous set of data collected in Sweden between 2011 and 2012. The original set of data was collected using strategic sampling^2^ procedures and in accordance with ethical guidelines produced by the Swedish Research Council (Gustafsson, Hermerén, & Petterson, 2011), which protects participants’ rights in all areas of the research process. The original set of data was structured around asking 30 young adults in Sweden about their choice of a healthy lifestyle based on eating organic food. The original data were planned to be analyzed using a narrative qualitative method, but they had been left unanalyzed. The original interviews, which lasted between 1.5 and 2 hours, were conducted in the participants’ homes, as their homes were perceived as the most relaxed environment in which to share their experiences. More specifically, the original interviews were structured around two questions: (1) Can you tell me about your family and the experience of your own food tradition, from childhood to present? And (2) what everyday experiences do you have when it comes to organic food? Although these two questions did not follow the recommended question asked by descriptive phenomenological psychological researchers, who would specifically ask for a description of a situation in which the participants experience a phenomenon, most of the interviews included a description of a situation in which the phenomenon was experienced. Following a critical reading of all the interview material, several descriptions were discovered to have a similar structure to a phenomenological interview. It seemed as if several participants had interpreted the first question as a description of a situation, actually referring to their lifespan as a situation. The second question had been interpreted by several participants as an opportunity to describe their experience of choosing a lifestyle based upon an organic diet. After several critical readings of several interviews, a decision was made that a descriptive phenomenological psychological analysis would be fruitful and scientifically rewarding, because there were three interviews particularly (see Giorgi, 2009, p. 198) of young adults that were congruent with the phenomenological selection criterion^3^. Hence, there was a possibility of conducting a phenomenological psychological study of young adults’ experience of choosing a lifestyle based upon an organic diet.

The three descriptions selected from the previously collected data set consisted of one female, aged 18, and two males, aged 26 and 33, hence providing some internal variation in terms of the age span of the young adults. The participants^4^ were recoded for this particular analysis and named Participant 1 (P1), Participant 2 (P2), and Participant 3 (P3).

### Method of data analysis

As the study sought to explore a human phenomenon as lived in everyday life, the descriptive, phenomenological psychological method (Giorgi, 2009) appeared a logical choice for data analysis. The main research question that guided the analysis was “What is the psychological meaning of young adults’ experience when choosing a healthy lifestyle based on an organic diet?” The procedures followed the four consecutive steps of the descriptive phenomenological psychological research method in which the method of phenomenological psychological reduction was utilized throughout the data analysis (Giorgi, 2009). More specifically, phenomenological psychological reduction is a partial reduction that is carried out by the researcher by reducing the objects of the participants’ experience to phenomena, while treating the participants’ acts as real (Giorgi, 2009, p. 98). The psychological reduction is sometimes referred to as the researcher mentally bracketing the existential assumptions, theories, hypotheses, and prejudices about the phenomenon in order to be more fully present to the psychological meanings as expressed by the participant (Giorgi, 1992, 2009). In other words, it is a way to stay closer and thus be more true to what is being expressed in the data (in terms of psychological meaning) by not engaging psychological theories, other ontological presuppositions, and so on. The steps of the research method are as follows: (1) The researcher reads the entire description in order to get a holistic view of the experience, (2) meaning units are identified, (3) data are explicated to the level of a phenomenological psychology, and (4) an invariant, psychological meaning structure is discovered and carefully described (Giorgi, 2009). There are no specific questions to keep in mind during the data analysis, but instead the phenomenological researcher is guided by the psychological meaning constituted in the expression of the other. However, if one would, for pedagogical reasons, construct a question that guided the presence, it would be something like “What are the psychological meanings being expressed by the participants in the context of the situation and in relation to the phenomenon?” By using phenomenological psychological reduction, one is adopting a phenomenological attitude, meaning that one is directed toward the intentional acts and objects as expressed by participants in the situation. Steps 3 and 4 also include the use of imaginative variations, in which general meanings are sought by raising the idiographic meanings to the eidetic and general level of a phenomenological human science (Giorgi, 2009). Thus, the task is then for the researcher to explicate and describe, using psychological reduction and imaginative variations, the general psychological meaning of the phenomenon.

## Results

Considering the empirical variations in terms of the experience of the one female and two male young adults, the phenomenological, eidetic analysis disclosed a general, psychological meaning structure of choosing a healthy lifestyle based on an organic diet. The results are presented as follows: (1) the general psychological structure of the phenomenon and the relationship between the constituents, and (2) the constituents and their empirical variations.

### The general psychological structure of the phenomenon and the relationship between the constituents

The lived psychological meaning of choosing a healthy lifestyle based on an organic diet is experienced by the young adult as an overall complexity in terms of discovering a sense of independence and self-regulation. The lived body is seen as the starting point and the vehicle of a special skill, in which life is explored through nutrients, flavors, and food texture. The exploration through this special skill creates an enlightened life strategy in terms of well-being and vitality. The perceived healthy lifestyle is constituted by a set of values and ethical standards that transcends the individuals themselves and includes the well-being and vitality of the next generation, including animals and nature. A narrative self is disclosed within the context of the choice of a healthy lifestyle that reflectively relates to, emerges from, and persists in one's emotional memories in relation to food and others.

#### Constituents and their empirical variations

The general psychological structure of the phenomenon as described in the previous subsection represents the main finding of the study in which the constituents can be seen in their interdependent relationships with each other. It is common practice in descriptive phenomenological psychological research reports to also include a subsection in which the constituents are explored independently of each other, although it is important to note that, from a descriptive phenomenological psychological perspective, constituents (as opposed to elements or themes) are never independent of each other (Giorgi, 2009). In this study, four constituents were identified that held together the eidetic, general psychological structure of the phenomenon. These were (1) the lived body as the starting point for lifestyle exploration, (2) a narrative self through emotional-relational food memories, (3) a life strategy for well-being and vitality, and (4) a personal set of values in relation to ethical standards.

#### Constituent 1: The lived body as the starting point for lifestyle exploration

The participants expressed satisfaction about having a certain ability to “listen to their own body signals,” indicating that their lived body was perceived as a starting point for their exploration of a healthy lifestyle. They felt that by paying more attention to the experience of their own body, they allegedly discovered a great need for (or lack of) certain essential nutrients and energy. By supplying the body with these nutrients, they experienced a sense of well-being. One could say that their experience was characterized by being able to be present to positive and negative information from the health status of their body. The information from the body was perceived as specific needs that had to be satisfied.

For example, the experience of Participant 1 (P1) was that her body was informing her about specific needs and what it wanted to avoid. She had been using these “bodily signals” that she had experienced to articulate to others a congruency between her body and her new, alternative, healthy lifestyle (based upon an organic diet). P1 experienced that her body was informing her about needs for both nutrients and specific foods that provided her with feelings of satisfaction and well-being. She also discovered that she experienced positive feelings when she explored the organic food with her fingers, making an experiential connection between touching the exterior texture of organic food and choosing her new healthy lifestyle. She perceived this to mean that she had the ability to experience the equilibrium between her body and her choice of food. Thus, she experienced a strong sense of connection between her lived body and organic food, indicating a positive meaning in terms of her well-being. In addition, she felt as if she could trust her experience in informing her how to live and what was healthy for her.

Similarly, Participant 2 (P2) also had experiences that were as if the body directed him toward the type of food that he needed to eat. He believed that when he felt certain cravings for certain foods (e.g., cravings for eggs), he had the ability to experience the lack of the empirical property (e.g., protein) connected with such foods. He became drawn to this type of experience and interpreted it as a certain ability that was previously unknown to him and that he wanted to learn more about. As a consequence, he became confident that his experience could provide him with the information to decide what vitamins and nutritious food he needed to eat at any given moment. This also established a sense of trust to provide a healthy lifestyle and well-being for himself. He also felt that he had become more aware of how his body reacted to the food and that this experience made him more aware that it is through his body that he discovers life. P3 then experienced the lived body as the starting point for exploring a healthy lifestyle based upon organic food. He claimed to experience positive feelings for organic food and related these positive feelings to his interpretation of organic food as “fresh” and as a choice of being healthy. In contrast, he experienced strong negative feelings toward nonorganic food, which he judged as wrong to eat, as tasting bad, and as an unhealthy choice.

Participant 3 (P3) described similar experiences in which he perceived his body as “signaling” his needs for specific foods. He interpreted these experiences as new and unfamiliar to him earlier in life. Furthermore, he felt as if his new perception provided him with a different and deeper understanding of what his body meant to him. He claimed that this made him understand himself better. For example, he felt that when he experienced tiredness, he tried to relate it to what he had just eaten and consequently made a connection to the type of energy that he needed. He felt that food could give him positive feelings and fill him with energy. He remembered that when he began to eat more colorful vegetables and fruit, it had a positive effect on him and provided him with a sense of awakening or vitality. Hence, the participants’ healthy lifestyle was experienced as self-regulated by the lived body's ability to explore the experiential need for certain foods.

#### Constituent 2: A narrative self through emotional-relational food memories

The second constituent was marked by the participants’ experience of forming a narrative self through emotional-relational food memories. In other words, a healthy lifestyle, based on eating organic food, was experienced as having an interdependent connection to positive childhood memories and to the development and formation of a self in young adulthood that has evolved over time.

P1 remembered growing up in a family where food had great importance and the family spent time and interest on cooking and socializing with food. Both her family and the opportunity to try new foods and flavors in different countries during childhood had influenced her perception of herself, and even her close relationship with food, her food choices, and cooking. She connected food with relationships to important people in her life, and the more she cooks with certain others, the closer a relationship she has with them. She remembered that she composed and cooked dinners and invited friends to share them with her as early as second grade, and she, along with her friends, arranged great parties where they invited other families. As before, when she now meets her close friends, she tries to cook food according to their needs and preferences. When meeting new people, she felt that she could not get the same close relationship through food with them, compared with people she knew earlier in life. Today, she tries to eat healthily, which she associates with organic food, vegetables, and fruit. Her first memory of organic food was when she developed an allergy that forced her to change the way she ate. She remembered that her mother bought her some organic apples that she experienced as fresh, delicious, and healthy.

As with P1, P2 remembered that food and cooking were important parts of his family community when he was a child. He remembers the variety of cuisines from the two families he lived with during his childhood, and he remembered that they had very different traditions and relationships with food. Apart from being with his own family, he also spent long periods of his childhood in the home of his nanny. His nanny's family lived in the countryside, grew much of their own food, and cooked food according to the season. He felt that they had new and sustainable ways of preparing and eating food, which differed from those of his own family. He remembered that they allowed him to be involved in the daily cooking at his nanny's home, and that together they brought different ingredients (root vegetables and eggs) from the food produced on the farm. He felt that these events gave him emotional food memories associated with his nanny. He also remembers what his nanny's family ate every day of the week, and how they cooked food at his nanny's place. In contrast, he had trouble remembering what he ate at home. What he had not forgotten was that dinner was always a major meeting place for his family. After a teenage period of his life, which mostly consisted of fast food, he tried exploring different foods and ways of eating together with friends. Today, food is very important to him and his new family. He emphasizes that food is an important part of the culture in which he lives, that his identity is constituted by his way of relating to food, and that it is due to the way his nanny related to food. Thus, P2 associates traditionally prepared meals with being in a meaningful relationship with a significant other.

Similarly, P3 also had an experiential connection between how he perceived his current lifestyle and early emotional-relational food memories. He remembered being at home, always having leafy vegetables at every meal and a lot of meat. He felt that the meals were composed and prepared with care by his mother but not always healthy. She gave him and his brother enough food and always three regular meals a day. Moving out as a teenager changed his way of eating and living, and he started to eat mostly junk food, drink large amounts of coffee, and also change his regular eating habits. He remembered that he did not take food seriously, that food did not really mean anything to him. His relationship with food was based solely on making him happy and satisfied, but he did not understand that, for example, breakfast was important for his well-being for the rest of the day. He also pointed out that during that period, he ate irregularly and often only one or two meals a day, and he felt that it affected his motivation and mood. Today, both his approach to food and his own way of life have changed. He feels that he has been inspired by his mother's early care through food and by deepening his relationship with his brother, who shares his approach to food. Together, they have started to gain more knowledge, in particular about organic food.

#### Constituent 3: A life strategy for well-being and vitality

The third constituent identified was characterized by the participants’ experience of a need to deliberately develop a strategy for a sustainable balanced life, marked by well-being and vitality. The experience of well-being was directly associated with gaining access to organically grown food that they could prepare and eat, providing feelings of great satisfaction. The experience of satisfaction enhanced their experience of vitality. Consequently, the acquired strategy was seen as a healthy and prosperous lifestyle that stimulated a sense of well-being, including caring for others.

For P1, choosing organic food meant that she had found a baseline for a balanced healthy lifestyle. For example, she had experienced positive emotions, feeling healthy, and the ability to show kindness to herself. Organic food had become a natural part of her life strategy and narration about herself and how she now feels and thinks about food and life. Organic food has given her a new awareness, which means that through her own choices she may affect her own well-being, which she had not previously experienced. She felt that her relationship to organic food is based on several factors, but one factor stands out: organic food provides her with a balanced, healthy lifestyle. P1 claims that organic food provides her with the opportunity to be exposed to a diet consisting of mainly vegetarian food and fruit that taste good and contain vitamins and other essential nutrients. The positive feelings that she experientially connects with organic food have made her choose organic food as constituting her healthy lifestyle of well-being and vitality.

P2 expressed intense positive feelings in terms of his choice of a lifestyle based upon an organic diet and how this choice has had an effect on his experience of feeling healthy and provided him with a new sense of vitality. He now experiences less need for excessive sleep and a stronger sense of life equilibrium and well-being. Another aspect of well-being was that he felt that certain types of food gave him positive emotions and created a special bond between himself and the food he prepared. Organic food provided him with a positive emotional bond to his own vitality and allowed him to take better care of himself. Leading a lifestyle based upon organic food has helped him in establishing a sustainable experience of serenity and in making important decisions based on a better sense of awareness.

P3 expressed that he found a healthy balance in life based on his relationship with food. This way to find balance is also an attempt to create self-acceptance and acknowledge his own needs. Choosing a lifestyle based upon organic food has turned into an existential issue for him and a way to show a real commitment to himself, which led him to become aware of how different choices affected his health. It was when there was a confrontation between incompatible demands and stressful life events that he slowly began to reevaluate his life. In connection with the reevaluation, he began to explore the importance of food and “energy,” resulting in his life now being more balanced. Today, he places great emphasis on the food he eats looking appealing and inviting, and he uses his senses in an active manner when he chooses food that he feels is good for him. In addition, he feels extra-strong positive emotions of joy when he has the ability and time to create a meal that gives him back his vitality. Giving priority to having time to cook healthy meals has become a qualitative measure of how his life works.

#### Constituent 4: A personal set of values in relation to ethical standards

The lived psychological meaning of a healthy lifestyle based upon an organic diet is also essentially constituted by a personal set of values in relation to ethical standards. The participants expressed their own values as a need to respect themselves, other people, animals, and nature.

For P1, the choice of organic food has become a way to show respect for herself, others, and the world and thus to provide her life with values consistent with her lifestyle. For her, respect means the right to decide for herself who she is and wants to be, and to let other people have their own self-determination about what they are thinking or doing without being negatively judged by other people. She feels that she wants to share her ethos with others, but not by telling them what to think or do, but instead by allowing them to make their own informed choices. She uses herself and the organic food she cooks and eats as a way to communicate to others a possibility to live a healthy lifestyle. She also shares the love of the food she prepares with other people around her, and she hopes that they will see that her choice of food has a value, tastes better, and is less toxic. She often feels challenged about her choice of a healthy organic lifestyle and that she constantly has to defend herself and her way of living. She feels that the criticism comes from people in both the past generation and her own generation and that such criticism makes her sad, worried, and constantly on guard. She is nevertheless willing to stick to her choice because she feels that it is right for her to be able to show respect for herself, other beings, and nature. She feels that organic food promotes a good lifestyle by showing respect for people as well as other creatures and the environment. Therefore, she links eating organic food to how she perceives her healthy lifestyle and how she defines herself as an ethical person.

P2 saw a healthy lifestyle as a direct link to be able to continue to live and to show respect and love for other people and animals. For him, it is important to have the Earth to live on, and that is best protected through a sustainable society based on the principle that everyone eats organic food. He feels that previous generations made the wrong choices in life by not taking into account that the generations who follow should also be able to exist on the Earth, which can give them both food and shelter. At the same time, he believes that every individual has their own right to make lifestyle choices without being challenged by others. For him, choosing organic food is making an active choice that takes into account future generations, including animals and nature. He sees food as both love itself and as a way to show concern for others, which means that it is particularly important that food is grown, handled, and prepared with care and caution. He prefers to treat the majority of all living beings with the same respect.

For P3, eating organic food means that he deliberately chose a sustainable healthy lifestyle that does not cause excessive environmental impact. It was when he was approaching his 30th birthday that he began to think about his life, what he ate, and how food is produced as he tried to find another way to live his life. He began to reevaluate himself and his existence, and through changing his way of thinking and feeling, he developed a greater understanding of his own role in the ecosystem and what his big-city, materialistic way of life really meant. It made him rethink how he used resources, and instead of buying more clothes, he started to use his money to buy food with higher quality and better nutrition. For him, organic food was a more complex situation than the actual eating; it was about his whole existence and what he and perhaps others will carry over to the next generation in the form of healthy eating and basic human values.

## Discussion

This discussion section begins with a brief critical discussion of the research methodology utilized in this study, followed by a critical dialog between the results and some relevant literature. An attempt is then made to suggest some conclusions that could act as the foundation for some plausible implications for the psychology of health and well-being. The section ends with suggestions for future research.

### Critical discussion about the method

In general, the descriptive phenomenological psychological method is limited by participants’ descriptions of their experience of the phenomenon under study. Also, some appearances and nuances of the phenomenon might have stayed concealed to phenomenological psychological description. Regarding the limitations just stated, this study was delimited by the use of phenomenological psychological reduction, imaginative variations, the description of psychological meaning, and presentational evidence as a source for eidetic certainty.

In addition, using previously collected data that had been gathered using a narrative qualitative approach might also have directed the overall psychological structure into a narrative phenomenological psychology. However, the phenomenon includes the aspect of “choice of a lifestyle,” which implies a narrative theme. In other words, it is doubtful that the data collection procedure had an essential impact on the discovery of the reported results, because the narrative aspect was part of the phenomenon. Also, the performance of a phenomenological analysis on interviews that have been gathered from a different qualitative perspective has been previously conducted and defended by Giorgi (1986). Phenomenological analyses of qualitative data collected from a different qualitative perspective could be fruitful if the perspectives are not completely dissimilar and “the initial context and research situation are kept in mind and respected” (Giorgi, 1986, p. 5).

The researchers employed Giorgi's (2009) selection criterion (i.e., that the participants must have had an experience of the phenomenon) to the present study. Because the selection was based upon previous material, it was a selection of previous interviews. The researchers critically examined the 30 interviews and selected several interviews in which the phenomenon was clearly present. Following Giorgi (2009), p. 198), three were analyzed in order to seek the general psychological structure of the phenomenon. Also, the use of phenomenological psychological reduction, in which theories (including narrative theories) are bracketed, and the use of eidetic, imaginative variation provide researchers with critical methodological tools to discover, explicate, and describe the general psychological structure of the phenomenon under study.

### Critical dialog with previous literature

There were some convergences between constituent 1 (i.e., “the lived body as the starting point for lifestyle exploration”) and findings from studies discussing the role of the body in how we approach the world. For example, in a consumer study conducted by Halkier (2001), food consumption is seen as a tension between a desire for a certain food and not wanting to eat it because of certain ingredients (e.g., artificial additives). From a phenomenological perspective, Halkier's (2001) study could be interpreted as indicating that we struggle with several different modes of our intentionality (i.e., we can have a desire for a food object while also being aware of some of its undesirable physical properties, e.g., artificial additives). However, the present study showed that by choosing a lifestyle based upon organic food, young adults can find a way to overcome such conflicting intentional acts of consciousness. In other words, the organic choice sets up a symbolic harmony between the properties of the food and the desire.

In another study, Ristovski-Slijepcevic, Chapman, and Beagan (2008) found that a person's bodily knowledge was as important as the scientific knowledge deciding what kind of nutrition he or she needed. The participants would state that they would, for example, crave a certain nutrient (e.g., protein). This is quite like the adoption of words from the physical sciences (e.g., “energy”) as used in meditative activities such as yoga, in which the experience of a closer connection to one's lived body also becomes possible (see, e.g., Morley, 2008, p. 158). In other words, one can suggest that a closer connection to our lived body becomes possible through a choice of organic food as a healthy lifestyle, similar to what occurs when we meditate.

Constituent 2 of this phenomenon (i.e., “a narrative self through emotional-relational food memories”) had certain similarities to previous health-related findings. For example, Devine (2005) found that people's food choices incorporate meaningful experiences together with thoughts, feeling, and actions (e.g., trajectories). They are developed cumulatively over the lifetime and are affected by experiences through the life span. For example, Devine (2005) states that the adolescent's transition to becoming an adult is an opportunity to try different identities as an eater. In addition, according to Croll, Neumark-Sztainer, and Story (2001), young people associate healthy food with fruit and vegetables, eaten in a situation “such as home” (p. 195) or together with the family. Hunt et al. (2011) found that young people value family meals and that they like to be activated and involved in food-related activities; at the same time, they use food to create a life separate from their families, by trying to come to terms with their identity, security, and independence. Hunt et al. (2011) also stated that even if there are situations in which family relationships are experienced as problematic, cooking and eating at home have positive significance in the lives of young people, indicating that there is a certain sense of congruency with constituent 2, especially in relation to significant others early in life (e.g., the family).

There were also some similarities between constituent 3 (i.e., “a conscious life strategy for well-being and vitality”) and some overall results of other studies on nutrition. Allicock et al. (2008) noted that health has a broader, more holistic meaning than just being physically well. For example, being healthy is to be adaptive and flexibly adjusted to changing circumstances, and to have mental and spiritual wellness. Furthermore, Harrison & Jackson (2009) discussed physical and emotional meanings that youth associate with healthy food such as increased energy and positive emotions like feeling happy, stress-free, and relaxed. Michaelidou and Hassan (2008) found that people who decided to eat organic food were more aware of their health, were also concerned about their state of well-being, and were more inclined to know how to improve and maintain their health and quality of life. Köpke (2005) found nutrient quality in relation to psychological and emotional well-being and concluded that the social effects from eating organic food were more important than a measurable contribution of a balanced diet to individual health. Köpke (2005) noted that this was an extended definition of nutrition, also covering the psychological effects of well-being based on knowledge related to ethical, environmental, and social values.

Constituent 4 (i.e., “a personal set of values in relation to ethical standards”) was similar to some aspects reported in consumer studies. For instance, studies by Stobbelaar et al. (2007) showed that adolescents considered organic food both healthy and environmentally friendly, particularly the characteristics of organic food related to personal interests and ethical preferences (e.g., animal welfare and environmental considerations). Michaelidou and Hassan (2008) noted that ethical motives might be part of a person's self-identity that is directly related to a particular behavior, which in turn positively affects the person's attitude toward organic food. In another study, Bisogni et al. (2005) found that providing meals for others meant taking care of and recognizing other people's needs.

The phenomenon of choosing a lifestyle based on an organic diet from the perspective of the young adult can provide us with psychological insights into embodied consciousness and its direction toward the natural world. There might be many different reasons behind making a lifestyle from the base of an organic diet; however, in living through its psychological meaning, it is an intricate congruency between the lived body and a narrative self. The strong ethical commitments directly connect one to an intersubjectivity of values and thus to a belonging to a group. Vitality and the reward of well-being cement the phenomenon as an experience of being part of nature.

## Conclusions, implications, and suggestions for future research

For the young adult, choosing a lifestyle based upon an organic diet constitutes a return to the natural world on a philosophical level, whereas on a psychological level it connects one to aspects such as identity, values, and well-being. One could suggest that the discovery of a lifestyle based on organic food provides the young adult with a sense of psychological stability in life that is congruent with one's history and perception of health. The lifestyle thus provides for vitality by relating one's being to the natural world (setting up a contrast to taking control over the natural world). But what does all this mean in terms of contributing to our understanding of health and well-being? One interpretation would be that the root of health and well-being is closer to us than we might think, that is, it is embodied and thus part of our lived experience. However, this does not mean that we should overthrow the natural scientific definitions of health and well-being and revert to a sense of traditionalism or mysticism. Instead, it could provide for a new venue of phenomenological, health-related research based on diets and the lived body.

One implication of this type of research is that we could become better at promoting a healthier lifestyle for the young adult if we are able to relate it as a lifestyle. Often, we seem to promote the adding of “good parts,” such as more vitamins or exercise, as a means to health and well-being. Perhaps we need to start early in life and establish, for example, healthy emotional memories of food that are congruent with our values and that could work as a psychological anchoring of a healthy lifestyle. Also, to facilitate a change of lifestyle is perhaps what is necessary in order for the psychological integration between identity and health to become possible in the first place. Nevertheless, more research is necessary in order to more fully understand the role of the lived body, especially in relation to the narrative aspects of the self. Such research provides us with the opportunity to dig deeper into the constitutive processes of a healthy lifestyle change based upon an organic diet. In addition, more research seems to be needed that has a focus on the connection between different food-related lifestyles of the young adult and psychological well-being.

## Conflict of interest and funding

There are no conflicts of interest. The study was supported by The Swedish Research Council Formas.

## Footnotes

1 Following Young et al. (2011) in this context, “young adult” refers to the age span between 18 and 35.

2 The 30 participants were recruited through requests for participation through various social media: Facebook, blogs, and digital newsletters with a national distribution and a focus on organic food. Social media are the information channels that young adults today are using to get instant knowledge. The inclusion criteria for participating in the study were being between 18 and 35 years old and having an interest in organic food. All who signed up and met the criteria were interviewed.

3 For a more extensive discussion on the selection criterion (i.e., the interviews contained in-depth descriptions of the phenomenon), see Giorgi (2009, p. 122). See also Giorgi (1986, p. 4–5) for the possibilities of conducting a phenomenological psychological analysis using data initially collected from a different qualitative perspective.

4 P1 is an 18-year-old woman who lives in a small town and is studying her final year in high school. P2 is a 26-year-old male, married, who is living in a small town; he works part-time and is at home part-time with his 2-year-old son. P1 is 33 years old and male; he lives in a big city and is both studying and working.

## References

[CIT0001] Adams W. W (2005). Ecopsychology and phenomenology: Toward a collaborative engagement. Existential Analysis.

[CIT0002] Allicock M, Sandelowski M, DeVellis B, Campbell M (2008). Variations in meanings of the personal core value “health”. Patient Education and Counseling.

[CIT0003] Bisogni C. A, Connors M, Devine C. M, Sobal J (2002). Who we are and how we eat: A qualitative study of identities in food choice. Journal of Nutrition Education and Behavior.

[CIT0004] Bisogni C. A, Jastran M, Seligson M, Thompson A (2012). How people interpret healthy eating: Contributions of qualitative research. Journal of Nutrition Education and Behavior.

[CIT0005] Bisogni C. A, Jastran M, Shen L, Devine C. M (2005). A biographical study of food choice capacity: Standards, circumstances, and food management skills. Journal of Nutrition Education and Behavior.

[CIT0006] Croll J. K, Neumark-Sztainer D, Story M (2001). Healthy eating: What does it mean to adolescents?. Journal of Nutrition Education.

[CIT0007] Devine C. M (2005). A life course perspective: Understanding food choices in time, social location, and history. Journal of Nutrition Education and Behavior.

[CIT0008] Devine C. M, Connors M, Bisogni C. A, Sobal J (1998). Life-course influences on fruit and vegetable trajectories: Qualitative analysis of food choices. Journal of Nutrition Education.

[CIT0009] Fischler C (1988). Food, self and identity. Social Science Information.

[CIT0010] Giorgi A (1986). A phenomenological analysis of descriptions of concepts of learning obtained from a phenomenographic perspective. Fenomenografiska Notiser 4.

[CIT0011] Giorgi A (1992). Description versus interpretation: Competing alternative strategies for qualitative research. Journal of Phenomenological Psychology.

[CIT0012] Giorgi A (2009). The descriptive phenomenological method in psychology: A modified Husserlian approach.

[CIT0013] Gustafsson B, Hermerén G, Petterson B (2011). God forskningssed.

[CIT0014] Halkier B (2001). Risk and food: Environmental concerns and consumer practices. International Journal of Food Science and Technology.

[CIT0015] Harrison M, Jackson L. A (2009). Meanings that youth associate with healthy and unhealthy food. Canadian Journal of Dietetic Practice Research.

[CIT0016] Hjelmar U (2011). Consumers’ purchase of organic food products: A matter of convenience and reflexive practices. Appetite.

[CIT0017] Hunt G, Fazio A, MacKenzie K, Moloney M (2011). Food in the family: Bringing young people back in. Appetite.

[CIT0018] Köpke U (2005). Organic foods: Do they have a role?. Forum of Nutrition.

[CIT0019] Magnusson M. K, Arvola A, Hursti U.-K. K, Åberg L, Sjöden P.-O (2003). Choice of organic foods is related to perceived consequences for human health and to environmentally friendly behaviour. Appetite.

[CIT0020] Merleau-Ponty M (1962). Phenomenology of perception (D. A. Landes, Trans.).

[CIT0021] Merleau-Ponty M (1964). The visible and the invisible.

[CIT0022] Michaelidou N, Hassan L. M (2008). The role of health consciousness, food safety concern and ethical identity on attitudes and intentions towards organic food. International Journal of Consumer Studies.

[CIT0023] Morley J (2008). Embodied consciousness in tantric yoga and the phenomenology of Merleau-Ponty. Religion and the Arts.

[CIT0024] Neumark-Sztainer D, Story M, Ackard D, Moe J, Perry C (2000). The “family meal”: Views of adolescents. Journal of Nutrition Education.

[CIT0025] Pellegrini G, Farinello F (2009). Organic consumers and new lifestyles: An Italian country survey on consumption patterns. British Food Journal.

[CIT0026] Ristovski-Slijepcevic S, Chapman G. E, Beagan B. L (2008). Engaging with healthy eating discourse(s): Ways of knowing about food and health in three ethnocultural groups in Canada. Appetite.

[CIT0027] Schifferstein H. N. J, Ophuis P. A. M. O (1998). Health-related determinants of organic food consumption in the Netherlands. Food Quality and Preference.

[CIT0028] Stobbelaar D. J, Casimir G, Borghuis J, Marks I, Meijer L, Zebeda S (2007). Adolescents’ attitudes towards organic food: A survey of 15- to 16-year old school children. International Journal of Consumer Studies.

[CIT0029] Sylow M, Holm L (2009). Building groups and independence: The role of food in the lives of young people in Danish sports centres. Childhood: A Global Journal of Child Research.

[CIT0030] Woodgate R. L, Leach J (2010). Youth′s perspectives on the determinants of health. Qualitative Health Research.

[CIT0031] Young R. A, Marshall S. K, Valach L, Domene J. F, Graham M. D, Zaidman-Zait A (2011). Transition to adulthood: Action, projects, and counseling.

